# Repurposing SGLT-2 Inhibitors to Target Aging: Available Evidence and Molecular Mechanisms

**DOI:** 10.3390/ijms232012325

**Published:** 2022-10-14

**Authors:** Rosalba La Grotta, Chiara Frigé, Giulia Matacchione, Fabiola Olivieri, Paola de Candia, Antonio Ceriello, Francesco Prattichizzo

**Affiliations:** 1 IRCCS MultiMedica, Polo Scientifico e Tecnologico, Via Fantoli 16/15, 20138 Milan, Italy; 2 Department of Clinical and Molecular Sciences, DISCLIMO, Università Politecnica delle Marche, Via Tronto 10/A, 60100 Ancona, Italy; 3 Center of Clinical Pathology and Innovative Therapy, IRCCS INRCA, 60100 Ancona, Italy; 4 Department of Molecular Medicine and Medical Biotechnologies, University of Naples Federico II, 80131 Naples, Italy

**Keywords:** sodium-glucose cotransporter 2 inhibitors, cardiovascular outcomes, mortality, diabetes, aging, inflammaging, IL-6, caloric restriction, nutrient-sensing pathways, senescence, inflammasome, COVID-19

## Abstract

Caloric restriction promotes longevity in multiple animal models. Compounds modulating nutrient-sensing pathways have been suggested to reproduce part of the beneficial effect of caloric restriction on aging. However, none of the commonly studied caloric restriction mimetics actually produce a decrease in calories. Sodium-glucose cotransporter 2 inhibitors (SGLT2-i) are a class of drugs which lower glucose by promoting its elimination through urine, thus inducing a net loss of calories. This effect promotes a metabolic shift at the systemic level, fostering ketones and fatty acids utilization as glucose-alternative substrates, and is accompanied by a modulation of major nutrient-sensing pathways held to drive aging, e.g., mTOR and the inflammasome, overall resembling major features of caloric restriction. In addition, preliminary experimental data suggest that SGLT-2i might also have intrinsic activities independent of their systemic effects, such as the inhibition of cellular senescence. Consistently, evidence from both preclinical and clinical studies have also suggested a marked ability of SGLT-2i to ameliorate low-grade inflammation in humans, a relevant driver of aging commonly referred to as inflammaging. Considering also the amount of data from clinical trials, observational studies, and meta-analyses suggesting a tangible effect on age-related outcomes, such as cardiovascular diseases, heart failure, kidney disease, and all-cause mortality also in patients without diabetes, here we propose a framework where at least part of the benefit provided by SGLT-2i is mediated by their ability to blunt the drivers of aging. To support this postulate, we synthesize available data relative to the effect of this class on: 1- animal models of healthspan and lifespan; 2- selected molecular pillars of aging in preclinical models; 3- biomarkers of aging and especially inflammaging in humans; and 4- COVID-19-related outcomes. The burden of evidence might prompt the design of studies testing the potential employment of this class as anti-aging drugs.

## 1. Introduction 

Caloric restriction (CR) has been recognized to halt the aging rate and increase the healthspan and lifespan in animal models for almost 50 years [1]. Recent data suggest that this effect may be also present in humans, albeit only preliminary evidence has been provided [2]. The search for the potential mediators of the beneficial effect of CR led to the discovery of nutrient-sensing pathways, suggested to be key drivers of aging in animal models [3]. Given the difficulty in maintaining a low-calorie regimen for long periods, alternative dietary and pharmacological strategies have been proposed [4]. For instance, a limited number of molecules have been suggested to reproduce the effect of CR on selected nutrient-sensing pathways, and have been thus termed CR mimetics. CR mimetics are mainly drugs interfering with glucose metabolism, e.g., metformin and 2-deoxyglucose, or compounds modulating the 5′ AMP-activated protein kinase (AMPK)-mammalian target of rapamycin (mTOR) pathway, e.g., rapamycin and resveratrol [5,6]. Many of these drugs have been suggested to improve lifespan and/or healthspan in animal models [6,7]. However, none of them have actually produced a loss of calories and, more importantly, only some of them were indeed reported to provide a benefit in terms of age-related, hard outcomes in humans [7,8]. 

Sodium–glucose cotransporter (SGLT)-2 is an integral membrane protein encoded by the *SLC7A2* gene, which transports the glucose across the cell membranes in the kidney. In particular, SGLT-2 promotes the reabsorption of 80–90% of the glucose filtered by the glomerulus and reuptake in the early proximal tubule [9]. SGLT-2 inhibitors (SGLT-2i) are a class of glucose-lowering drugs promoting glucose elimination through urine. The most studied SGLT-2i are all members of the class of “gliflozins”, with dapagliflozin, empagliflozin, and canagliflozin being the most prescribed [10]. Mechanistically, these molecules might present subtle differences in terms of selectivity for SGLT-2 over SGLT-1 inhibition, but the observed effects on hard outcomes are largely overlapping for this class [11]. The glycosuria induced by these drugs produces a loss of about 200 kcal/day, thus promoting a consistent weight loss despite a compensatory hyperphagic response [12]. The burden of glucose shunted into urine results in a progressive shift in fuel utilization toward fatty substrates and in a marked ketogenesis at the systemic level, both sustained by decreased insulin levels and an increased glucagon\insulin ratio [13,14]. These metabolic adjustments largely resemble those induced by CR [15]. 

Multiple trials and large observational studies have shown a protective effect of SGLT-2i against major adverse cardiovascular (CV) events, hospitalizations for heart failure, CV-related death, all-cause mortality, and renal outcomes in patients with diabetes [9,10,16,17]. Of note, SGLT-2i have also been demonstrated to improve heart failure-related endpoints, kidney outcomes, and possibly also all-cause mortality in patients without diabetes [18,19,20]. A tempting hypothesis underpinning such observations could be that the mimicry of CR is the main culprit of the beneficial effect of SGLT-2i [21,22,23]. As a corollary, considering that aging is the main risk factor of all these conditions [24], SGLT-2i might promote healthspan and lifespan by ultimately targeting the aging process itself. To support this postulate, here we summarize the available data relative to the effect of SGLT-2i on: 1- animal models of healthspan and lifespan; 2- selected molecular pillars of aging in preclinical models; and 3- biomarkers of aging, especially inflammaging, in humans.

## 2. Effect of SGLT-2i in Animal Models of Lifespan and Healthspan

The first report relative to the effect of SGLT-2i on animal lifespan showed that TA-1887, a highly potent and selective SGLT-2i not employed in clinical practice, was able to increase the mean lifespan of cachectic *db/db* mice subjected to a long-term high-fat diet [25]. Mechanistically, treatment with SGLT-2i was associated with a marked reduction of inflammation, oxidative stress, and cellular senescence in visceral adipose tissue, three key phenomena known to be associated with the aging process [26,27]. Of note, such effects were observed also when comparing SGLT-2i with insulin [25], suggesting that an improved glycaemic control might not be the main driver of the beneficial effect of SGLT-2i. 

These results were then substantiated and extended in a rigorous study exploring the effect of SGLT-2i on the longevity of genetically heterogeneous mice not exposed to a diabetogenic environment. In this context, canagliflozin, administered from the seventh month of age until death, extended the median survival of male mice by 14% and increased by 9% the age for 90th percentile survival, an effect not observed in female mice [28]. A histopathological study conducted in these mice revealed that the use of SGLT-2i was able to lower the incidence or the severity of cardiomyopathy, glomerulonephropathy, arteriosclerosis, hepatic microvesicular cytoplasmic vacuolation (lipidosis), and adrenal cortical neoplasms, thus likely affecting also healthspan beyond lifespan [29]. These effects are consistent with the benefit demonstrated by this class in multiple clinical trials, which have shown a reduction in terms of hard outcomes relative to heart failure, kidney disease, and, more recently, also hepatic steatosis [9,10,16,17,30]. In another study, a treatment of 30-month-old mice with canagliflozin was associated with an improvement of exploratory and locomotor activity in male but not female mice, suggesting that the healthspan-promoting effects of SGLT-2i can be observed also with late treatment [31].

In summary, these studies suggest that SGLT-2i consistently promote lifespan and healthspan in mice, albeit the effect might be confined to males only, an aspect deserving further investigation. 

## 3. Effect of SGLT-2i on the Pathways of Aging 

### 3.1. Effect on Nutrient-Sensing Pathways

Nutrient-sensing pathways are known drivers of aging. Many of the genes regulated by nutrients have been shown to regulate lifespan in animal models and have been thus termed “nutrient-sensing longevity genes” [32,33]. 

Among the most relevant nutrient-sensors, mTOR, AMPK, and sirtuin-1 (SIRT1) pathways are key and intertwined modulators of aging [34]. mTOR, a fundamental regulator of cellular growth and metabolism, is a serine–threonine kinase of the phosphatidylinositol-3-OH kinase (PI(3)K)-related family. mTOR activity is controlled by nutrients and hormones and is mediated by the formation of two complexes: mTOR complex 1 (mTORC1) and mTOR complex 2 (mTORC2), both linked to the aging process [35]. Insulin and growth factors stimulate mTORC1 through PI(3)K and protein kinase B (AKT) kinase signalling, whereas AMPK has an inhibitory effect [35,36]. In turn, AMPK is a sensor of the energetic status of the cell, activated in response to the increase of the AMP/ATP ratio and playing a crucial role in regulating whole-body energy balance [37]. AMPK can also activate SIRT-1, a nicotinamide adenine dinucleotide (NAD)-responsive deacetylase regulating a large range of metabolic genes, ultimately promoting fatty acid oxidation and ketogenesis [38]. 

Several experimental evidences have supported the idea that SGLT2 inhibitors modulate these nutrient-sensing pathways. For instance, the renoprotective effects of SGLT-2i has been attributed to the ability of these drugs to increase ketone levels, which in turn blunt mTORC1 hyperactivation that occurs in diabetic kidney disease [39]. Similarly, other studies have found an increased autophagic flux associated with mTOR inhibition in the kidneys of obese or diabetic mice treated with SGLT-2i [40,41], an effect also observed in vitro in human renal proximal tubular cells exposed to high glucose [40]. Furthermore, the modulation of mTOR pathways was also observed in multiple tissues beyond the kidneys. Indeed, old mice treated with canagliflozin have shown a reduction in the phosphorylation levels of the ribosomal protein S6, a downstream target of mTOR, in the hypothalamus and hippocampus, an effect associated with a reduction of age-associated hypothalamic gliosis and a decreased inflammatory cytokine production by microglia [31]. 

SGLT-2i have been suggested to blunt mTOR activity also via an indirect effect on AMPK [42]. In particular, multiple studies have shown that SGLT-2i can restore the AMP/ATP balance in favour of AMP to activate AMPK, a mechanism that has been suggested to mediate the beneficial effect of these drugs on the heart structure and microvasculature, as observed in two different murine models [43,44]. On the other hand, AMPK does not seem mandatory for the metabolic benefits of SGLT-2i in animal models, while fibroblast growth factor (FGF) 21, a peptide mediating some of the benefits of fasting, explains only part of the effect [45,46]. 

In summary, data from preclinical models suggest a consistent modulation of nutrient-sensing pathways by SGLT-2i in multiple tissues. This modulation may be mediated by a direct effect of SGLT-2i on the activity of key nutrient-sensing proteins, such as mTOR and AMPK, but also generated by a systemic metabolic and hormonal reshaping occurring at the organismal level upon SGLT-2i administration. 

### 3.2. Effect on Oxidative Stress and Mitochondrial Dynamics

Mitochondria are dynamic organelles, with a central function in energy production, metabolic tuning and apoptosis initiation. For this reason, mitochondria are finely regulated by processes of fission and fusion to continuously adapt their shapes and sizes and respond to extracellular stimuli and intracellular needs [47,48]. Among the hallmarks of aging, the progressive loss of this well-organized machinery causes a reduction in mitochondrial membrane potential and respiratory capacity, generally associated with an enhancement of oxygen free radicals (ROS) [32,49]. Many age-related conditions, such as neurodegenerative, metabolic and renal diseases, among others, are indeed also characterized by a mitochondrial impairment [50]. Notably, when SGLT2i were evaluated in pathological conditions different from diabetes mellitus, beneficial outcomes were reported on the mitochondrial dynamics, although the specific molecular mechanism remained unexplored [43,48,51]. Furthermore, the renal protective effects of SGLT2i in both human renal proximal tubular cells and in obese and diabetic mice were consistently traced back to the capability of these molecules to re-establish mitochondria morphology and function. In particular, Takagi S. and colleagues have registered the restoration of physiological levels of mitofusin 2 (Mfn2), and optic atrophy 1 (Opa1), relevant factors implicated in mitochondrial fusion; while Lee YH et al. have observed how the expression of another mitofusin protein (Mfn1), and also of dynamin-related protein 1 (Drp1, implicated in mitochondrial fission), were also restored by SGLT2i treatment [40,51]. Another mechanism potentially implicated in the SGLT2i effect on mitochondria includes AMPK [40]. Indeed, Empagliflozin was shown to delay microvascular endothelial cell senescence and improve angiogenesis, thus lowering cardiac injury, in a mouse model of diabetes through the activation of AMPK and the consequent inhibition of mitochondrial fission via suppression of Drp1 mitochondrial recruitment [43]. In another study, the cardioprotective effects of SGLT2i were linked to an improvement of mitochondrial functionality via normalization of mitochondrial membrane potential, Ca^2+^ homeostasis, decreased ROS and RNS (reactive nitrogen species) production, a recovered ADP/ATP ratio, and balance between fusion- and fission-related proteins [52]. A similar rescue of the mitochondrial function, biogenesis and dynamics was also reported in the atrial cells of diabetic rats [53]. Moreover, Mizuno M. and colleagues observed that Empagliflozin was able to prevent the reduction of mitochondria number and size, known to occur in the heart of diabetic mice in subsequence of an infarction [54]. 

The capacity of SGLT2i to regulate mitochondrial functionality has also been related to the neuroprotective action of these inhibitors. As a relevant example, Dapagliflozin was able to improve brain mitochondrial function and insulin signalling, thus preventing neuronal dysfunction and cognitive decline in obese insulin-resistant rats, with a marked reduction of insulin resistance and brain oxidative stress [55]. 

### 3.3. Effect on Cellular Senescence and Inflammatory Pathways 

Low-grade inflammation is a key driver of aging. The progressive and systemic development of a sterile pro-inflammatory status that occurs during aging has been termed inflammaging, and is ascribable to a range of possible mechanisms [56,57]. Among them, the progressive accrual of senescent cells with a pro-inflammatory phenotype, the chronic stimulation and activation of immune cells, and a range of metabolic alterations mainly promoted by overnutrition are held to be major fuels of this chronic inflammatory environment [58,59]. 

SGLT-2i have been suggested to consistently ameliorate all these phenomena. A number of studies have suggested that SGLT-2i are able to decrease the rate of senescence in the kidneys, but also in other organs, e.g., the heart, as shown in multiple mice models of diabetes [60,61,62]. Of note, albeit the effect of hyperglycaemia on the development of senescence is well-established [63], the effect of SGLT-2i on this phenotype appears to be mediated by an improvement of antioxidant defences induced by ketone elevation and not simply by an amelioration of glycaemia, since glimepiride, a sulphonylurea promoting glycaemic control through insulin secretion, was unable to reproduce the anti-senescence effect of SGLT-2i [61]. In addition, selected reports have also shown a direct effect of SGLT-2i against senescence in vitro. In particular, albeit SGLT-2 is mainly expressed in the kidneys, a consistent expression of this protein has also been shown in other tissues, with specific stimuli promoting its increase [64,65]. Indeed, angiotensin II is able to induce senescence and parallelly augment the abundance of SGLT-2 in endothelial cells, an effect blunted by the co-treatment with SGLT-2i [66]. Similarly, both dapagliflozin and empagliflozin were able to counteract the pro-senescence effects induced in endothelial cells by ponatinib, a chemotherapeutic with known cardiovascular toxicity [67]. Overall, these data suggest that SGLT-2i are endowed with anti-senescence properties, which have been observed with different pro-senescence stimuli, and that could be either indirect, i.e., mediated by the abovementioned hormonal and metabolic rearrangements, or even directly mediated by the inhibition of SGLT-2 at the cellular level. In the case of a confirmed expression of this protein in non-renal cells, the hypothesized direct anti-senescence effect of SGLT-2i would not be surprising since it is well recognized that senescent cells have a high metabolic activity and rely mainly on glucose to sustain their overactive secretory program, which underlies their pro-inflammatory phenotype [68]. Thus, reducing the glucose influx in senescent cells is likely to result in an attenuation of their inflammatory program, or even in a selective inability to survive the milieu in which they live. 

Despite the large range of self- and non-self-molecules triggering the chronic activation of the innate immune system, the molecular pathways suggested to drive inflammaging are limited [57]. One key pathway suggested to mediate the age-related pro-inflammatory drift is the NLRP3 (NLR family pyrin domain containing 3) inflammasome platform. The NLRP3 inflammasome is activated by multiple pathogen- or damage-associated molecular patterns (PAMPs or DAMPs) and consists in the assembly of the multi-protein complex NLRP3-apoptosis-associated speck-like protein (ASC) C-terminal caspase-recruitment domain (CARD) promoting the splicing of pro-IL-1β and pro-IL-18 precursors into their active forms through caspase-1 cleavage [69]. Different murine models have demonstrated that the genetic inhibition of this pathway is able to: 1- increase healthspan and lifespan in normal mice; 2- promote metabolic health and immune status in both aged and obese mice; and 3- prevent cardiac dysfunction in aged mice [70,71,72,73]. Multiple studies have suggested an inhibition of the NLPR3 inflammasome and a reduced secretion of IL-1β after treatment with SGLT-2i in murine models [74,75,76]. Mechanistically, it was previously demonstrated that the switch from glycolysis to ketogenesis deactivates the inflammasome in immune cells and reduces immunopathology [77]. Thus, the SGLT-2i-induced ketonemia likely reproduces this phenotype [22]. A pilot trial substantiated this framework by comparing macrophages collected from SGLT-2i treated patients and patients treated with sulphonylureas to reach the glycaemic equipoise. Macrophages from SGLT-2i treated patients showed a reduced secretion of IL-1β, an effect mediated by high β-hydroxybutyrate levels and by the decrease in serum insulin [78]. Similarly, we demonstrated that patients on therapy with SGLT-2i have a less severe systemic inflammatory status when compared to patients on other therapies and matched for a large range of variables, an effect possibly mediated by the reduced levels of insulin and uric acid, which trigger or boost inflammatory responses in macrophages and endothelial cells [79]. 

Overall, these data suggest that SGLT-2i inhibit the accumulation of senescent cells and blunt the activation of the NLRP3 inflammasome in multiple tissues, two phenomena held to be major drivers of aging. The metabolic switch towards ketonemia, the decrease in insulin and uric acid levels, and a putative direct role of SGLT-2 in senescent cells are all likely intermediate phenomena explaining these observations. 

## 4. Effect of SGLT-2i on Biomarkers of Aging and Inflammaging in Humans

As mentioned above, inflammaging is progressively being recognized as a key driver of aging, promoting the development of virtually all age-related diseases. Among the large range of pro-inflammatory mediators increasing during aging, interleukin (IL)-6 is probably the most studied cytokine in relation to age-related outcomes [80]. Indeed, circulating IL-6 levels have been associated with the development of frailty, diabetes, cardiovascular events, all-cause mortality, and other age-related diseases in multiple cohort studies [81,82,83,84,85]. The burden of evidence prompted the definition of IL-6 as the “cytokine for gerontologists” [86]. 

SGLT-2i have been demonstrated to reduce IL-6 levels in three different studies [79,87,88]. In particular, all three studies have compared patients on treatment with SGLT-2i with matched patients on other therapies and with similar glycaemic controls, thus suggesting that the anti-inflammatory effect of SGLT-2i is additive to their ability to lower glycaemia. In addition, the same studies have shown that high-sensitivity C-reactive protein (hs-CRP), a protein secreted by the liver in response to inflammatory stimuli, is not affected by SGLT-2i treatment [79,87,88]. On the contrary, the analysis of the plasma samples from a trial comparing the effect of the SGLT-2i canagliflozin with that of a sulphonylurea demonstrated a reduction of a plethora of other pro-inflammatory and pro-fibrotic mediators, i.e., TNF receptor (TNFR) 1, matrix metalloproteinase 7, and fibronectin 1 in patients treated with the SGLT-2i [87]. Of note, TNFR-1 and 2, monocyte chemoattractant protein (MCP)-1, and kidney injury molecule-1 (KIM-1), another pro-inflammatory molecule previously linked to the development of kidney events, were demonstrated as intermediate risk factors of the renal benefit provided by canagliflozin in the CANVAS trial [89]. In this respect, it must be emphasized that canonical risk factors, e.g., HbA1c, LDL-cholesterol, body weight, and blood pressure, have been suggested to mediate only a minor part of the benefit provided by SGLT-2i on cardiovascular and kidney outcomes in clinical trials [90], suggesting that non-canonical mediators such as inflammatory molecules might be of relevance to explain the benefit of these drugs. To our knowledge, no study has explored IL-6 or other inflammatory mediators as intermediate molecules explaining the benefit of SGLT-2i on cardiovascular outcomes or all-cause mortality. 

Beyond inflammation, the use of SGLT-2i is associated with an improvement of the markers of renal function [91] and heart failure [92]. SGLT-2i are also hypothesized to promote hemopoiesis [93], albeit it is unclear whether the increase in haematocrit results from a stimulation of erythropoietin secretion, or simply reflects a phenomenon of haemoconcentration. Whatever the case, markers such as creatinine and NT-pro-BNP are known to be progressively affected by aging [94,95] and SGLT-2i can at least partially revert this trend. A different, recently proposed biomarker of aging trajectories is miR-21, which is increased during aging and in age-related diseases [96]. Of note, a 3-month treatment with empagliflozin was recently reported to restore the blood circulating quantities of this miRNA in frail older adults with heart failure and diabetes mellitus; the study also demonstrated that this effect was specific for the SGLT-2i, compared with treatment with insulin, or also metformin in the same patients [97].

In summary, the above-described literature is compatible with the hypothesis that SGLT-2i treatment may promote a pro-longevity response through a pleiotropic beneficial effect on diversified molecular targets and biological processes linked to unhealthy aging. 

## 5. Effect of SGLT-2i on COVID-19 Outcomes

Older age, male sex, and the presence of age-related diseases are the strongest risk factors for severe outcomes in patients with coronavirus disease (COVID)-19, the disease driven by the infection of SARS-CoV-2 [98]. All three of these conditions are characterized by a more pervasive status of inflammaging, which predisposes patients with these features to develop excessive inflammation coupled with an inefficient immune-mediated viral neutralization, two phenomena held to drive severe COVID-19 outcomes [99,100,101]. 

Given the abundance of data suggesting a protective effect of SGLT-2i against cardiovascular and kidney diseases, common complications also of patients with COVID-19 [102], these drugs were explored as a possible strategy to halt the progression of COVID-19 to severe forms. Observational data has supported this hypothesis, since patients on chronic therapy with SGLT-2i are protected by COVID-19-related mortality, as shown in a population study with data from a large UK database [103]. However, a clinical trial testing the effect of acute SGLT-2i administration in patients diagnosed with non-severe COVID-19 and previously not on therapy with this class showed only a non-significant trend towards a protection against a composite outcome of prevention (i.e., the time to new or worsened organ dysfunction or death) [104]. However, subgroup analyses have revealed that a significant protection against this same outcome was observed in males but not females [104], an intriguing result, considering the abovementioned observations relative to the selective pro-longevity effect of SGLT-2i in male mice and the acquired knowledge relative to the sexual-dimorphism in human immune system aging [105].

## 6. Concluding Remarks and Future Prospects

CR mimetics have been suggested as potential routes to promote longevity and postpone or limit the development of age-related diseases. Aging still does not represent an indication for intervention for regulatory agencies, albeit “ageing associated decline in intrinsic capacity” has been listed as an ICD’s diagnostic category in the latest ICD-11 release [106]. The planned TAME (targeting aging with metformin) trial will test for the first time whether metformin, a glucose-lowering drug mainly promoting insulin sensitivity in the liver, is able to decrease the incidence of age-related diseases in patients with at least one age-related disease and without diabetes [107]. In the case of success, the results of this trial might eventually disclose age as a modifiable risk factor, albeit clinical models to demonstrate an anti-aging effect for any drug are still far from being fully implemented. Whether this kind of trial will become more common in the future is unknown. If so, we postulate that SGLT-2i are the perfect candidate for such a study, as has also been suggested by others [108].

**Figure 1:**
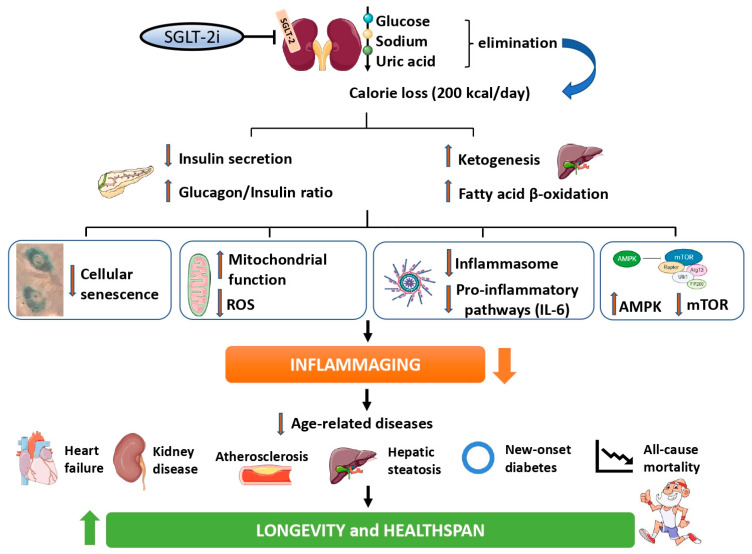
**A graphical summary of the framework hypothesizing an anti-aging effect of SGLT-2i.** SGLT-2i induce the elimination of glucose, promoting a loss of roughly 200 Kcal per day, a phenomenon followed by a decrease in insulin levels and an increased glucagon/insulin ratio, which all together promote a shift from glycolysis towards fatty acids utilization and ketogenesis. This systemic metabolic and hormonal reshaping has been suggested to attenuate a range of pathways held to drive aging, including cellular senescence, the accumulation of ROS and mitochondrial dysfunction, the activation of inflammatory pathways including the inflammasome platform, and the overactivity of key nutrient-sensing proteins such as mTOR. These effects might cumulatively ameliorate inflammaging at the systemic level, and a lower degree of low-grade inflammation could benefit a large range of age-related outcomes, including cardiovascular diseases, kidney disease, diabetes development, hepatic steatosis, and all-cause mortality, ultimately promoting healthy longevity.

SGLT-2i induce the loss of glucose and calories, triggering a metabolic reshaping closely resembling the effect of CR. This hesitates in a marked inhibition of a number of pathways held to drive aging, e.g., mTOR, cellular senescence, and the inflammasome, consequently ameliorating inflammaging at the systemic level. In turn, a lower low-grade inflammatory status could result in a tangible decrease in the incidence of age-related diseases, overall promoting healthy longevity (summarized in Figure 1 and resumed in Table 1).

Solid evidence from multiple trials has already demonstrated the ability of SGLT-2i to decrease the incidence of cardiovascular events, hospitalizations for heart failure, progression of kidney disease, hepatic steatosis, new-onset diabetes, and all-cause mortality [9,10,16,17,109]. Of note, many of these effects, including those on all-cause mortality, were observed also in patients without diabetes, but with at least one age-related morbidity, e.g., heart failure or kidney disease [18,19,20]. Thus, the rationale and the data available to test the effect of SGLT-2i on any age-related, hard endpoint in a broad population is consistent. On the other hand, some concerns relative to the general safety, and in particular on the effect of SGLT-2i on bone density, lower limb complications, ketoacidosis, and bladder cancer, have been raised, albeit many of these warnings have been debunked [110,111]. In addition, it must be considered that SGLT-2i are available on the market within relatively few years, which renders it unfeasible to extrapolate their long-term effects on age-related outcomes. For instance, their effect on new overall cancer diagnosis seems neutral at present [112], but with the limitation that all the studies conducted thus far have a follow-up of only few years, and have thus little relevance for the study of the incidence of oncological diseases. If SGLT-2i tangibly affect the aging rate, then a decreased rate of selected age-related malignancies, e.g., breast and prostate cancers, should be expected. Thus, longer-term observational studies are needed before employing SGLT-2i in a wide population for prevention purposes. Finally, the use of SGLT-2i is associated with an increased incidence of genitourinary infections, commonly attributed to their glycosuric effect [113]. However, preliminary evidence suggests that the incidence or the outcomes linked to other infectious diseases such as pneumonia might be decreased by the use of SGLT-2i [114], an observation that might reflect their benefit on the aging immune system, but that requires additional knowledge. Finally, more data relative to the effect of these drugs in men vs. women should be thoroughly explored, given the preliminary evidence from mice studies and human patients with COVID-19 showing a putative, selective benefit for the male sex.

In summary, here we propose a framework postulating that part of the benefit provided by SGLT-2i is ascribable to their ability to recapitulate the effect of CR and thus counteract aging pathways. Beyond the academic interest, if the plethora of benefits (and the limited burden of serious side effects) demonstrated in the clinical trials is confirmed in long-term studies and is appliable to a large population, then SGLT-2i might be tested as a potential strategy to postpone age-related diseases and promote healthy longevity.

## Figures and Tables

**Table 1:** Summary of the main findings relative to the effects of SGLT-2i on: 1- lifespan and healthspan in mice; 2- aging pathways in animal models; 3- markers of inflammaging in humans; and 4- the incidence of age-related diseases in patients with or without diabetes.

Effect	Findings	References
Effect on healthspan and lifespan in mice	Sglt2 inhibitor TA-1887 increased the mean lifespan of cachectic *db/db* mice under a long-term high-fat diet	Sugizaki T et al., 2017 [25]
	Canagliflozin extended median survival by 14% and increased by 9% the age for 90th percentile survival of male mice, improving also the mice’s healthspans	Miller RA et al., 2020 [28] Snyder JM et al., 2022 [29]
	Canagliflozin improved exploratory and locomotor activity in 30-month-old male but not female mice	Jayarathne HSM et al., 2022 [31]
Effect against aging pathways in animal models	Ketone bodies-mediated inhibition of mTOR and modulation of other nutrient-sensing pathways (AMPK-SIRT1)	Zhou H et al., 2018 [43] Tomita I et al., 2020 [39]
	Rescue of normal mitochondrial fusion/fission dynamics and normalization of mitochondrial membrane potential with attenuated oxidative stress	Durak A et al., 2018 [52] Lee YH et al., 2019 [40]
	Inhibition of cellular senescence in multiple tissues and models	Madonna R et al., 2020 [62] Kim MN et al., 2021 [61]
	Inhibition of the NLPR3 inflammasome	Leng W et al., 2016 [75]
Effect against inflammaging in humans	Macrophages from SGLT2-i treated patients have a reduced secretion of IL-1β compared with patients on other therapies with similar glycemic control	Kim SR et al., 2020 [78]
	Patients treated with SGLT-2i have lower circulating levels of IL-6, compared with patients on other therapies and comparable glycemic control	Garvey et al., 2018 [88] Heerspink et al., 2019 [87] La Grotta et al., 2022 [79]
	Treatment with SGLT-2i reduce the circulating levels of a plethora of pro-inflammatory and pro-fibrotic markers, which mediate part of the renoprotective effects of the drugs	Sen T et al., 2021 [89] Tye SC et al., 2021 [90]
Effect against age- related diseases in humans	SGLT-2i reduce the incidence of major adverse cardiovascular events, hospitalizations for heart failure, CV-related death, all-cause mortality, and renal outcomes in patients with type 2 diabetes	Wang C et al., 2019 [91] McGuire DK et al., 2020 [83] Li CX et al., 2021 [17]
	SGLT-2i reduce the incidence hospitalizations for heart failure, CV-related death, all-cause mortality, and renal outcomes also in patients without diabetes but with heart failure or kidney disease	McMurray JJV et al., 2019 [19] Heerspink HJL et al., 2020 [18] Silverii GA et al., 2021 [20]
	Treatment with dapagliflozin decrease the incidence of new-onset type 2 diabetes in patients with chronic kidney disease or heart failure	Rossing P et al., 2022 [109]
	SGLT-2i improve hepatic steatosis in patients with diabetes	Reviewed in Scheen AJ et al., 2019 [30]
